# A cross-sectional study to compare levels of psychiatric morbidity between young people and adults exposed to violence in a large urban center

**DOI:** 10.1186/s12888-016-0847-0

**Published:** 2016-06-07

**Authors:** Denisse Jaen-Varas, Jair de Jesus Mari, Evandro da Silva Coutinho, Sérgio Baxter Andreoli, Maria Ines Quintana, Marcelo Feijó de Mello, Rodrigo Affonseca Bressan, Wagner Silva Ribeiro

**Affiliations:** Departamento de Psiquiatria, Universidade Federal de São Paulo, Rua Borges Lagoa, 570, São Paulo, SP 04038-000 Brazil; Health Service and Population Research Department, King’s College London, Institute of Psychiatry, London, United Kingdom; Fundação Oswaldo Cruz, Escola Nacional de Saúde Pública, Rio de Janeiro, Brazil; London School of Economics and Political Science, Personal Social Services Research Unit, London, United Kingdom

## Abstract

**Background:**

Teenagers and young adults are more exposed to violence and traumatic events than adults, and these factors can be associated with mental disorders. This paper aims at investigating whether young people are more exposed to violence and traumatic events and to compare pattern of mental disorders with adults.

**Methods:**

Cross-sectional study using the Composite International Diagnostic Interview, conducted between 2007 and 2008 with a randomly selected sample of 15 to 75 year-old residents of São Paulo, Brazil.

**Results:**

Two thousand five hundred thirty-six participants were divided into two groups: 1096 (43.2 %) young people (15 to 24 years), and 1440 (56.8 %) adults (25 to 75 years). 12-month exposure to traumatic events was higher among young people (32.1 % vs. 20.6 %; *p* < 0.001). Assaultive violence was reported by 13.4 % of young people and 8.6 % of adults (*p* = 0.012); 20.1 % of young people and 13 % of adults reported suffering other injury or shocking events (*p* < 0.001); sudden death/life threatening illness of a close person was declared by 6.1 % of young people and 3.2 % of adults (*p* = 0.017). Prevalence of alcohol related disorders was higher among young people (5.4 % vs. 2.5 %; *P* = 0.032); depressive disorders were more prevalent among adults (9.0 % vs. 4.7 %; *P* = 0.004). Alcohol related disorders were associated to assaultive violence among young people (OR = 3.4; 95 % CI = 1.36 to 8.52; *p* = 0.004) and adults (OR = 2.38; 95 % CI = 1.23 to 4.61; *p* = 0.002). Phobic/anxiety disorders were associated to other injury or shocking events among young people (OR = 1.28; 95 % CI = 0.67 to 2.44; *p* = 0.025). Major depressive disorder was associated to assaultive violence among young people (OR = 2.27; 95 % CI = 1.09 to 4.74; *p* = 0.004) and adults (OR = 1.28; 95 % CI = 0.85 to 1.93; *p* = 0.009).

**Conclusion:**

Exposure to violence and traumatic events was higher among young people. Alcohol related disorders, depression and phobic/anxiety disorders were significantly higher among young people exposed to traumatic events. Despite the study design, high exposure to violence and traumatic events in this age group can be considered important factors in triggering mental disorders in this vulnerable age period.

## Background

Adolescence is an important stage in human development, and it is characterized by decreased incidence of disease and good average health conditions. However, risky behaviors and life styles that may have long term effects throughout adult life [1–3] are a common during adolescence. Brain development is not complete until the age of 25. Therefore, important hormonal and neurobiological changes in specific brain regions and cell populations [4, 5] are still ongoing during adolescence and early adulthood. Moreover, several behavioral changes occur during adolescence that can be triggered and/or intensified by contextual factors, such as community, school and workplace characteristics, as well as peers and family influences [4, 6–10]. Adolescence can, therefore, be defined as a turbulent period characterized by impulsivity, sensitivity to peer influence, intensified emotional reactivity, novelty seeking, mood dysregulation, and risky behaviors [2, 5, 11], which may increase exposure to violence and other traumatic events [5, 12]. Adolescence is also a peak period for clinical onset of most mental illnesses [5, 6, 13].

Available data suggests that exposure to violence is more prevalent among adolescents and young adults, in comparison to other age groups [2, 14, 15]. The World Health Organization’s World Report on Violence and Health [15], for example, estimates that homicide rates in the year 2000 in the age group ranging from 10 to 29 was 9.2 per 100,000 individuals worldwide. According to the same report, in Brazil, 39.7 % of deaths among young people between the ages of 15 and 24 resulted from homicides, whereas, among adults, homicides accounted for 1.8 % of deaths. Among young people, only 26.4 % of deaths result from natural causes, whereas 90.1 % of deaths among adults are attributed to natural causes [15]. Available evidence on exposure to violence, however, predominantly rely on homicide rates, and few studies effectively probe youngster exposure to types of violence that are common in urban centers [16–18].

Population-based studies have identified violence as a major risk factor for the development of mental disorders [18–20], mainly when experienced at an early age and/or when there is an accumulated exposure during the cycle of life [9, 12]. Exposure to situations such as domestic violence or conflict [21], negative life events [22], persistent threat, bullying [23], parental loss or abuse [21] can have a permanent effect across the person’s life [24]. The maturation of the brain during adolescence makes this age group particularly vulnerable to the development of mental disorders [1, 6, 14, 20, 25–27], especially because young people are possibly more vulnerable as a result of their greater exposure to violence. It has been proposed that violence can negatively affect brain development, leading to changes to the structure (reduce to volume in the hippocampus, corpus callosum, cerebellum, and a smaller prefrontal cortex.) and chemical activity of the brain (altering neurogenesis, migration, synaptogenesis, and neurochemical differentiation) [6, 8, 11, 28]. Evidence has shown that such changes to the brain’s structure and chemical activity are associated to several mental health problems [6, 8, 29, 30]. This article aims to study how exposure to violence and other traumatic events affects the mental health of young people and adults living in a large urban centre in Brazil. In short, the following hypothesis are tested: a) exposure to violence and traumatic events is greater in young people than adults; and b) young people and adults exposed to violence and traumatic events have different patterns of mental disorders.

## Methods

This article is based on a secondary analysis of data from a one-phase cross-sectional survey that was designed to estimate the prevalence of exposure to traumatic events and of mental disorders in the general population. A detailed account of the study design, sampling strategy and data-collection procedures is provided elsewhere [20]. Briefly, data used in this article was collected in the city of Sao Paulo, which is the largest urban centre in Brazil. The city has over 11 million inhabitants. It is the major industrial, commercial and financial centre in the country, and presents high levels of socioeconomic inequality and violence.

The study was carried out in 2007–2008, and it included a representative sample of residents aged 15 to 75 years, through a multilevel stratified sample strategy. The stratification was based on the homicide rates as follow: firstly, the 96 administrative areas in the city were ranked according to their homicide rates, and then grouped into six strata. Secondly, all census sectors within each stratum were mapped, and then a number of census sectors were randomly selected within each stratum. In the third stage, 43 households were randomly selected within each selected census sector. Finally, in each selected household, one dweller aged between 15 and 75 years was randomly selected based on the Kish’s method [20, 31].

Variables of interest were assessed through the Composite International Diagnostic Interview, version 2.1 (CIDI 2.1) [20, 32–34], which was applied at participants’ households by trained lay interviewers. To assess exposure to violence and other traumatic events, authors added 20 new types of events to the original 11-event list in the CIDI 2.1 to include experiences that are common in Brazilian urban centres [20]. Lifetime and 12-month exposure to traumatic events were assessed. CIDI 2.1 diagnosis of mental disorders was based on Diagnostic and Statistical Manual of Mental Disorders, 4^th^ Edition (DSM-IV). Three diagnoses were selected to study the association between traumatic events and mental disorders in young people and adults: a) alcohol related disorders (Alcohol hazardous use, Alcohol dependence); b) major depressive disorders; and c) phobic/anxiety disorders (panic disorder, specific phobia, social phobia, agoraphobia, generalized anxiety disorder and obsessive-compulsive disorder). Therefore, adolescents and young adults are thought to be at greater risk of being exposed to violence and other traumatic events [12, 17].

### Statistical analysis

Prevalence estimates and confidence intervals were corrected according to sampling weights in order to adjust for differences in the probability of participant selection due the study design. The Sample was stratified into two groups, for every analysis: young people (ages 15–24) and adults (ages 25–75). The cut-point (24 years) was defined on the bases that, according to the literature, transition into adulthood is not fully complete before the age of 25 years [4, 6, 9], and, therefore, biological, social and cultural changes that define adolescence are still ongoing. Such changes could lead to specific vulnerabilities related to impulsivity and risk-taking behaviors that are common among adolescents and young adults [2, 5, 11]. Sample characteristics were described as analysis of frequency. Comparisons between age groups in relation to variables of interest were conducted by means of 2x2 contingency tables. Statistical significance of differences between groups were estimated through Pearson’s Chi Square test, adjusted for sampling weights.

As cumulative exposure to traumatic events tends to increase overtime, it would be expected prevalence of lifetime exposure to be higher among adults than young people as a function of age. Therefore, in order to assess between-groups differences in exposure to violence within a comparable timeframe, we considered only events that occurred in the last 12 months in order to verify whether exposure to traumatic events was higher among young people than adults.

When assessing the effect of exposure to traumatic events on psychiatric diagnoses, only traumatic events that happened more than 12 months prior to the interviews were included in the analyses as independent variables, whereas 12-month estimates of psychiatric disorders were defined as dependent variables. This modelling allowed for the establishment of temporal relationship between exposure to traumatic events and diagnoses in order to avoid reverse causality. The effect of exposure to traumatic events on psychiatric diagnoses was assessed through bivariate and multivariate analyses of association, which were based on the comparisons of proportions of diagnoses among those exposed and those not exposed to traumatic events. We excluded posttraumatic stress disorder (PTSD) because exposure to traumatic events is a mandatory diagnostic criterion, meaning that those who did not report exposure to traumatic events are not eligible to be diagnosed with PTSD.

To test the hypothesis that patterns of association between traumatic events and mental disorder would be different for young people and adults, analyses were stratified in these two age groups, and conducted in three steps: first, a bivariate association between each traumatic event and each diagnostic was estimated through 2x2 contingency tables. In a second step, multivariate logistic regression models were performed to control the effects of demographic characteristics (gender, marital status, education, occupational situation and migration history) as possible confounding factors. For each diagnosis entered as a dependent variable, the other two diagnoses were also entered in the logistic regression model as independent variables to control the effect of potential comorbidity. In these analyses, traumatic events were grouped into three binary variables (*yes* or *no*) that represent the three most relevant types of traumatic experiences according to the literature [20, 35–37]: a) assaultive violence; b) other injury or shocking events, which include indirect exposure to violence and accidents; and c) sudden death/life threatening illness of a close person, defined as a family member, close friend and/or romantic partner. All three types of traumatic events were entered together in the analyses, in order to control for the potential overlapping between them. In the two first steps, analyses were run separately for each age group. In the final step, the logistic regression models were repeated using the entire sample and including an interaction term between traumatic events and age group to assess the statistical significance of differences between Odds Ratios across the two age groups.

## Results

Table 1 presents the sample’s demographic characteristics. The final sample comprised 2536 participants, corresponding to 84.5 % response rate. The sample was composed of 1096 (43.2 %) young people aged 15 to 24, and 1440 (56.8 %) adults aged 25 to 75. Proportion of females was greater in both groups. More adults than young people were married or lived with a partner (63.9 % vs. 25.9 %; *p* < 0.001). Among young people, the proportion of those with no or low education (0–4 years) was significantly lower than among adults (2.3 % vs. 24 %; *p* < 0.001). More adults than young people were working at the time the study was done (62.6 % vs. 50.1 %; *p* < 0.001). Proportion of migrants was greater in the adult group (55.0 % vs. 28.0 %; *p* < 0.001). As can be seen in Table 2, prevalence of alcohol related disorders was greater in young people than in adults (5.4 % vs. 2.5 %; *P* = 0.032), whereas major depressive disorder was more prevalent among adults as compared to young people (9.0 % vs. 4.7 %; *P* = 0.004). No differences were detected in the prevalence of phobic/anxiety disorders.

**Table 1:** Socio-demographic characteristics of the sample

	15–24	25–75	*P* value
Gender			
Male	227 (43.3)	276 (41.6)	0.576
Female	869 (56.7)	1164 (58.4)	
Marital status			
Single	363 (72.5)	354 (18.4)	<0.001
Married/cohabiting	130 (25.9)	1337 (63.9)	
Separated/divorced	8 (1.2)	220 (10.7)	
Widowed	2 (0.5)	122 (7.0)	
Education (years of school)			
0–4	15 (2.3)	573 (24.0)	<0.001
5–8	155 (28.3)	551 (24.9)	
9–12	303 (60.1)	651 (34.1)	
13 or more	30 (9.3)	257 (17.0)	
Occupational status			
Currently unemployed	0 (0.0)	0 (0.0)	
Currently employed	246 (50.1)	1313 (62.6)	<0.001
Migration history			
Being a migrant	154 (28.0)	1231 (55.0)	<0.001

**Table 2:** Prevalence of 1-year DSM-IV mental disorders and exposure to violence and other traumatic events

	15–24	25–44	*P* value	OR (95 % CI)
One-year mental disorders				
Alcohol related disorders	26 (5.4)	64 (2.5)	0.032	0.6 (0.4–0.92)
Phobic/anxiety disorders	74 (15.8)	352 (17.3)	0.543	1.21 (0.93–1.6)
Major depressive disorder	32 (4.7)	190 (9.0)	0.004	1.52 (1.04–2.21)
Assaultive violence	59 (13.4)	165 (8.6)	0.012	0.67 (0.5–1.0)
War experience	0 (0.0)	0 (0.0)	0	0 (0–0)
Being attacked without weapon	8 (1.8)	16 (0.8)	0.142	0.5 (0.2–1.2)
Being attacked with weapon	15 (3.1)	44 (2.3)	0.435	0.72 (0.4–1.35)
Being kidnapped, or held captive	1 (0.1)	1 (0.1)	0.961	0.24 (0.01–4.1)
Fast kidnap	1 (0.1)	0 (0)	0.035	NA^a^
Torture/terrorism	2 (0.7)	0 (0)	0.014	NA^a^
Death threats	12 (3.1)	43 (2.1)	0.308	1 (0.46–1.68)
Conflict between gangs/drug dealers’	0 (0.0)	5 (0.3)	0.360	NA^a^
Rape	0 (0.0)	0 (0.0)	0	NA^a^
Sexual molestation	0 (0)	2 (4.92–04)	0.504	NA^a^
Being beaten-up by parents/relatives	7 (1.3)	7 (0.4)	0.075	0.24 (0.1–0.71)
Being beaten up by an intimate partner	6 (1.4)	20 (0.8)	0.313	0.82 (0.33–2.05)
Being beaten up by anyone else than family/partner	3 (0.3)	3 (0.2)	0.541	0.24 (0.05–1.22)
Having one’s house broken into while at home	17 (2.4)	25 (1.0)	0.057	0.52 (0.23–1.21)
Blackmailing telephone calls	13 (3.1)	55 (2.9)	0.925	1.05 (0.59–1.88)
Other injury or shocking events	106 (20.1)	270 (13.0)	0.001	0.6 (0.45–0.73)
Car/motorcycle accident	11 (2.3)	22 (1.0)	0.074	0.5 (0.24–1)
Accidents other than car/motorcycle	1 (0.1)	7 (0.5)	0.159	1.73 (0.2–14.7)
Fire, flood, natural disaster	2 (0.7)	11 (0.9)	0.745	1.4 (0.27–6.92)
Witnessing someone being killing or injured	32 (7.0)	58 (2.6)	<0.001	0.43 (0.27–0.69)
Witnessing bank robbery	2 (0.6)	13 (0.9)	0.640	1.61 (0.34–7.63)
Witnessing a shoot-out or being victim of stray bullet	18 (3.6)	32 (1.7)	0.030	0.43 (0.22–0.83)
Witnessing domestic violence during childhood	3 (0.3)	3 (0.3)	0.764	0.25 (0.066–0.91)
Having one’s house broken into while not at home	6 (0.7)	27 (1.3)	0.155	1.12 (0.42–3)
Seeing or touching a corpse	38 (6.8)	90 (4.1)	0.040	0.6 (0.4–0.82)
Witnessing atrocities, slaughter, massacre	6 (1.1)	20 (1.0)	0.824	0.82 (0.35–1.94)
Human made disaster	2 (0.7)	5 (0.3)	0.335	0.62 (0.14–2.7)
Witnessing crime organizations’	4 (0.4)	13 (0.3)	0.600	0.8 (0.3–2.44)
Sudden death/life threatening illness of a close person	93 (7.3)	130 (4.1)	0.013	0.6 (0.4–0.93)
Sudden unexpected death of a close person	30 (6.1)	73 (3.2)	0.017	0.6 (0.37–0.92)
Child with life threatening illness or injury	7 (1.3)	22 (0.9)	0.587	0.8 (0.35–1.7)
Any traumatic events	158 (32.1)	428 (20.67)	0.001	0.6 (0.46–0.73)

^a^NA = OR and 95 % CI not available as number of cases in at least one of the categories equal 0 (zero)

Table 2 also presents the prevalence of exposure to violence and other traumatic events. Exposure to traumatic events in the 12 months before to the interview was significantly higher among young people (32.1 % vs. 20.6 %; *p* < 0.001). Exposure to the three types of traumatic events in the 12 months before the interview was greater among young people than adults: exposure to assaultive violence was reported by 13.4 % of young people and 8.6 % of adults (*p* = 0.012); 20.1 % of young people reported suffering other injury or shocking events, as opposed to 13 % of adults (*p* < 0.001); sudden death/life threatening illness of a close person was declared by 6.1 % of young people and 3.2 % of adults (*p* = 0.017). Young people were more prone to car and motorcycle accidents than adults though not statistically significant (2.3 % vs. 1.0 %, *p* < 0.074), and more likely to witness someone being killed or injured (7.0 % vs. 2.6, *p* < 0.001).

Findings from the logistic regression models are presented in Table 3. As a result of the logistic regression models, through which the association of traumatic events and mental disorders was stratified by age groups (young people and adults) and controlled for potential confounding factors, we found the following associations: Alcohol related disorders were associated with assaultive violence among young people (OR = 3.4; 95 % CI = 1.36 to 8.52; *p* = 0.004) and adults (OR = 2.38; 95 % CI = 1.23 to 4.61; *p* = 0.002). Among adults, alcohol related disorders were also associated with other injury or shocking events (OR = 1.51; 95 % CI = 0.74 to 3.05; *p* = 0.030).

**Table 3:** Multivariate logistic regression model of the association between exposure to traumatic events and mental disorders

	Assaultive violence							
	15–24 years				25–75 years			
	No	Yes	O.R (95 % CI)	P	No	Yes	O.R (95 % CI)	P
Alcohol related disorders	8 (2.9)	18 (9.0)	3.4 (1.36–8.52)	0.004	13 (1.2)	51 (4.0)	2.38 (1.23–4.61)	0.002
Phobic/anxiety disorders	12 (3.8)	13 (6.8)	1.68 (1.01–2.78)	0.120	56 (7.5)	122 (10.5)	1.23 (0.94–1.61)	0.001
Major depressive disorder	12 (3.2)	20 (7.1)	2.27 (1.09–4.74)	0.004	63 (8.0)	127 (9.8)	1.28 (0.85–1.93)	0.009
	Other injury or shocking events							
	15–24 years				25–75 years			
	No	Yes	O.R (95 % CI)	P	No	Yes	O.R (95 % CI)	P
Alcohol related disorders	6 (1.9)	20 (7.3)	1.39 (0.44–4.38)	0.253	9 (1.4)	55 (3.4)	1.51 (0.74–3.05)	0.030
Phobic/anxiety disorders	4 (2.5)	21 (6.4)	1.28 (0.67–2.44)	0.025	29 (5.3)	149 (10.7)	1.31 (0.95–1.79)	<0.001
Major depressive disorder	5 (2.3)	27 (6.1)	2.36 (0.51–10.85)	0.114	33 (5.6)	157 (10.3)	1.65 (1.04–2.62)	0.001
	Sudden death or illness of a close person							
	15–24 years				25–75 years			
	No	Yes	O.R (95 % CI)	P	No	Yes	O.R (95 % CI)	P
Alcohol related disorders	20 (6.2)	6 (3.6)	0.62 (0.22–1.72)	0.518	32 (2.9)	32 (2.9)	1.11 (0.65–1.88)	0.215
Phobic/anxiety disorders	14 (4.5)	11 (5.9)	1.15 (0.70–1.91)	0.155	79 (7.5)	99 (11.4)	1.35 (1.06–1.71)	0.003
Major depressive disorder	19 (4.1)	13 (6.1)	1.17 (0.53–2.55)	0.345	92 (7.4)	98 (11.0)	1.03 (0.71–1.5)	0.120

Phobic/anxiety disorders were associated with other injury or shocking events among young people (OR = 1.28; 95 % CI = 0.67 to 2.44; *p* = 0.025). Among adults, these disorders were associated with exposure to assaultive violence (OR = 1.23; 95 % CI = 0.94 to 1.61; *p* = 0.001), other injury or shocking events (OR = 1.31; 95 % CI = 0.95 to 1.79; *p* = <0.001), and sudden death/life threatening illness of a close person (OR = 1.35; 95 % CI = 1.06 to1.71; *p* = 0.003).

Major depressive disorder was associated with exposure to assaultive violence among young people (OR = 2.27; 95 % CI = 1.09 to 4.74; *p* = 0.004) and adults (OR = 1.28; 95 % CI = 0.85 to 1.93; *p* = 0.009). The same condition was associated with other injury or shocking events in adults (OR = 1.65; 95 % CI = 1.04 to 2.62; *p* = 0.001).

The logistic regression analyses also show that female gender is associated with a lower likelihood of alcohol-related disorders among adults (OR = 0.35; 95 % CI = 0.19 to 0.63; *p* = 0.001); with a higher likelihood of anxiety disorders among youth (OR = 3.08; 95 % CI = 1.70 to 5.58; *p* < 0.001) and among adults (OR = 2.57; 95 % CI = 1.83 to 3.62; *p* < 0.001); and with a higher likelihood of depressive disorder among youth (OR = 5.97; 95 % CI = 2.27 to 15.71; *p* < 0.001), and among adults (OR = 3.0; 95 % CI = 1.99 to 4.69; *p* < 0.001). Education level among adults, expressed as number of years of schooling, was negatively associated with alcohol related disorders (OR = 0.92; 95 % CI = 0.86 to 0.99; *p* = 0.018), and with anxiety disorders (OR = 0.97; 95 % CI = 0.94 to 1.0; *p* = 0.039).

## Discussion

To the best of our knowledge, this is the first study that provides a comprehensive assessment of exposure to violence and other traumatic events among young people. Exposure to traumatic events in the 12 months before to the interview was significantly higher among young people (32.1 % vs. 20.6 %). Overall, young people were highly exposed to other injury and shocking events (20.1 %), assaultive violence (13.4 %), and sudden death/life threatening illness of a close person (6.1 %). Thus, our results confirm that exposure to violence and other traumatic events is greater among young people than adults. It has been proposed that youths have a higher risk of facing violence worldwide [2, 14, 38, 39]. However, most of the evidence on violence exposure is based on homicide rates [15, 39], which is recognized that homicide is one of the most comparable and accurate indicators for measuring violence [40]. Nonetheless, the World Health Organization recognizes that homicides represent only a fraction of overall violence, and suggests that more precise data of non-fatal violence must be gathered [15].

Our results validate the idea that psychiatric morbidity patterns are different among young people and adults. Phobic/anxiety disorders were the most prevalent diagnosis in both groups. Alcohol related disorders were the second most frequent diagnosis among young people, and were almost twice more prevalent among young people than adults (5.3 % vs. 2.9 %), whereas depression was twice more prevalent among adults than young people (9.0 % vs. 4.7 %). The higher prevalence of alcohol-related disorders among the youth in our study supports the assumption that harmful use of alcohol among young people living in urban centres of the low- and middle-income countries is very frequent. It has been suggested that young people usually experiment with alcohol to a great extent at this stage due to bio-psychosocial changes they experience [19, 20], as well as due to their natural drive to search for novelty [3]. It has also been proposed that risk factors such as family history of alcoholism, disturbed childhood behaviour, neuropsychological and/or developmental problems, poor relationships with colleagues and family, as well as unfavourable and violent neighbourhoods might interact individually or in group with genetic vulnerability to increase the incidence of alcohol abuse among young people. Some of the potentially negative consequences of abusive consumption of alcohol among adolescents include decreased self-control and increased risk behaviours. Use of Alcohol has been shown to be the leading cause of injuries, traffic accidents, violence and premature deaths around the world [2, 12, 14].

The higher prevalence of depression among adults may be a result of the usually later onset of this disease [19, 20], which results in depressive disorder being more prevalent in later stages of life [17, 41].

Our logistic regression models showed that the only demographic characteristic associated with psychiatric disorders among the youth was female gender, which was associated with an increased risk of anxiety and depression. Among adults, female gender was associated with a lower likelihood of alcohol-related disorders, and with an increased risk of anxiety and depression. Higher education level was also related with a lower likelihood of alcohol-related disorders and anxiety among adults. Both gender and education have consistently been found to be associated with the prevalence of mental disorders [42–44].

Additionally, we also tested the hypothesis that patterns of association between traumatic events and psychiatric morbidity would be different among young people and adults. Results showed that assaultive violence was associated with alcohol related disorders and major depressive disorder in young people and adults. Among young people, other injury or shocking events were associated with a higher probability of developing phobic/anxiety disorders, whereas the same type of event in adults was associated with an increase in the three psychiatric disorders under comparison. These results suggest that, whereas both groups are equally affected by the exposure to assaultive violence, the impact of other injury or shocking events seems to be greater among adults than among young people. One possible explanation relies on the cumulative effect of traumatic experiences over time, which may undermine one’s ability to cope with adversity [11, 17]. This may be due to the sensitization effect, which leads individuals previously exposed to traumatic events to have a greater responsiveness to subsequent stressors [2, 9, 41, 44].

The study’s cross-sectional design does not allow to discard reverse causality in the association between traumatic events and mental disorders. Evidence suggests that exposure to violence and other traumatic events favour the development of mental disorders [4]. However, mental disorders, particularly those associated with use of alcohol and drugs, may increase the risk of victimization [6, 7]. To minimize the possibility of reverse causality, statistical models included only traumatic events that occurred more than 12 months before the interviews, whereas psychiatric diagnoses in the last year were included in the analysis as dependent variables. Another potential limitation is that results were based on the CIDI 2.1, which is a standardized questionnaire that may not be accurate due to memory bias, besides being conducted by lay interviewers. Moreover, due to Brazilian heterogeneity, results may not be generalizable to the entire country, particularly to small and medium cities, where socioeconomic patterns and life styles may differ significantly from larger urban centres.

## Conclusions

This study provides evidence that young people are more exposed to violence and other traumatic events than adults, thus developing strategies to reduce violence amongst young people and to provide care for exposed victims in this age group is paramount.

A number of interventions to reduce vulnerability among adolescents and young adults has been tested. For instance, in Australia, Canada, the United Kingdom, and the United States of America (USA), it was shown that raising the minimum legal drinking age and having graduated licensing policies for teen drivers can reduce harmful use of alcohol, traffic accidents and criminality [12, 24], whereas in the USA, Fonagy and colleagues tested a manualized school psychiatric consultation program and the Creating a Peaceful Learning Environment, which is a manualized psychodynamic intervention, and concluded that both interventions were effective in reducing children’s experience of aggression and victimization, when compared to no intervention [23].

The results also suggest that the impact of exposure to violence on mental health is greater among adults, providing evidence that combating violence against young people could significantly reduce psychiatric morbidity later in life.

### Ethics approval and consent to participate

The study protocol was approved by the Research Ethics Committee of the Federal University of São Paulo, including Informed Consent Forms, data collection questionnaires, participants’ recruiting procedures, interview procedures and actions to protect privacy, integrity and rights of participants. Participants were interviewed only after signing the informed consent forms. For participants who were between 15 and 17 years of age, a legal guardian signed the consent form.

### Consent for publication

Not applicable

### Availability of data and materials

Data and materials are available upon request to Wagner Ribeiro, PhD. (W.Silva-Ribeiro@Ise.ac.uk).

## Abbreviations

CIDI 2.1: Composite International Diagnostic Interview, version 2.1
DSM-IV: Diagnostic and Statistical Manual of Mental Disorders, 4th Edition
PTSD: posttraumatic stress disorder
OR: Odds Ratio
P: Value P
CI: confidence interval
USA: United States of America
FAPESP: State of São Paulo Funding Agency
CNPq: National Research and Technology Development Council
CAPES: Coordination for Improvement of Faculty Personnel
PEC-PG: Students-Agreement Program Postgraduate
